# A Novel Adsorbent of Attapulgite & Carbon Composites Derived from Spent Bleaching Earth for Synergistic Removal of Copper and Tetracycline in Water

**DOI:** 10.3390/ijerph20021573

**Published:** 2023-01-15

**Authors:** Yuxin Ke, Xiaoli Zhu, Shaocheng Si, Ting Zhang, Junqiang Wang, Ziye Zhang

**Affiliations:** 1 College of Urban and Environmental Science, Northwest University, Xi’an 710127, China; 2 Shaanxi Key Laboratory of Earth Surface System and Environment Carrying Capacity, Northwest University, Xi’an 710127, China; 3 Xi’an Jinborui Ecological Tech. Co., Ltd., Xi’an 710065, China

**Keywords:** tetracycline, copper, synergistic removal, attapulgite & carbon composites, adsorption mechanisms

## Abstract

Simultaneously eliminating tetracycline (TC) and copper (Cu-II) from wastewater was investigated by applying a novel adsorbent fabricated by transforming spent bleaching earth (SBE) into attapulgite & carbon composites (A&Cs). Pyrolysis temperature for A&Cs preparation exhibited a positive effect on Cu(II) adsorption, while the AC500 possessed the greatest performance for TC remediation. Interestingly, a synergistic effect instead of competitive adsorption occurred between Cu(II) and TC under the combined binary system, as both TC and Cu(II) adsorption amount on A&C500 increased more than that in the single system, which could be mainly attributed to the bridge actions between the TC and Cu(II). In addition, hydrogen bonding, ᴨ-ᴨ EDA interaction, pore-filling and complexation exerted significant roles in the adsorption process of TC and Cu(II). In general, this study offered a new perspective on the regeneration of livestock and poultry industry wastewater polluted with antibiotics and heavy metals.

## 1. Introduction

Antibiotic and heavy metal pollutants are increasingly detected in water due to the continuous development of the livestock and poultry industry in China. Especially, manure wastewater has posed serious threats to ecological and human health due to the overuse of feed additives and antibiotics during the breeding process [1,2]. It was estimated that more than 162,000 tons of antibiotics in China were consumed per year, of which about 48% were applied for animal husbandry [3]. As a broad-spectrum antibiotic, tetracycline (TC) is widely used in animal disease prevention and treatment. However, approximately 50–80% of TC tends to be discharged into the environment after metabolism since animals usually contain an extremely low metabolic efficiency for TC [4,5]. Existing studies have found that the maximum concentrations of tetracycline (TC), chlortetracycline (CTC) and oxytetracycline (OTC) in typical aquaculture wastewater were 129.3, 25.4 and 32.7 μg/L in China, respectively, higher than pig wastewater in the United States (where the TC, CTC and OTC were 2.860, 2.740 and 3.140 μg/L, respectively) [6,7]. In addition, heavy metals (e.g., copper) were also widely applied as feed additives in fodder to accelerate animal growth. Nevertheless, these additives usually tend to contain more copper content than animals demand, coupled with lower digestibility in poultry, so most copper as a result is released into the environment along with metabolites [8,9,10]. Duan and Feng (2021) found that Cu, Zn and Cr could be extensively detected in the manure sample and sewage sludge from the Yanmenguan Cattle Herbivorous Livestock Area of China, while the Cu in swine manure accounted for a relatively high potential ecological risk from agricultural use, according to the value of Nemerow’s synthetic pollution index [11]. Exposure to excessive Cu(II) inhibits plant growth and destroys the ecological balance, further accumulating in the human body through the food chain to cause kidney damage [12]. Therefore, considering the current situation that both heavy metals and antibiotics have been detected simultaneously in water, more attention and effort should be paid to the synergistic elimination of Cu(II) and TC from aquaculture wastewater.

To date, various methods have been proposed to reduce or eliminate antibiotic and Cu(II) residues in aquaculture wastewater, such as biodegradation, chemical oxidation, photo-degradation and adsorption [13,14,15,16]. Among these, the adsorption method has provoked higher expectations due to its excellent performance, high efficiency, low cost and environmental-friendly nature [17,18]. Various adsorbents have been prepared from different biomass such as agricultural waste, municipal sewage, livestock manure, food residues, etc. [19]. As industrial waste from oil refineries, however, spent bleaching earth (SBE) has not been paid sufficient attention to in resource utilization. In 2019, there were more than 430 tons of SBE produced, while the residues of oil attached to SBE after the bleaching process severely limit its adsorption performance, even causing a hidden danger of emitting bad smells or combusting spontaneously [20,21,22]. The transformation of SBE into attapulgite & carbon composites (A&Cs) under pyrolysis might provide a potential method for harmless treatment, as residual oil in SBE and other impurities would be carbonized. In addition, the obtained A&Cs could also act as an adsorbent for the elimination of TC and Cu(II) since the release of oil residues might re-open the inside pores and introduce extra surface functional groups. However, the efficiency of A&Cs for the single and synergistic removal of Cu(II) and TC remains unclear, as these two pollutants with totally different properties usually co-exist in the wastewater.

Therefore, SBE was selected as raw material to prepare a novel adsorbent of A&Cs, and the main objectives of this study are to (1) evaluate the effects of applied pyrolysis temperature on the characteristics and adsorption performance of A&Cs; (2) use the optimal ACs to test the variation in adsorption performance for TC and Cu(II) under single and binary combined pollutant systems; (3) illuminate the adsorption mechanisms between A&Cs and TC/Cu(II). The conclusions from this research are expected to offer theoretical support for the treatment of SBE in a sustainable way and provide new material for the remediation of TC and Cu(II) from livestock wastewater.

## 2. Materials and Methods

### 2.1. Synthesis of Absorbent

The SBE in this study was collected from Xi’an Bangqi Oil & fat Co., Ltd. (Xi’an, China). The obtained samples were dried at 105 °C and then milled into particles of less than 0.15 mm. Next, SBE was pyrolyzed at 300 °C, 500 °C and 700 °C for 120 min with a heating rate of 5 °C/min in the muffle furnace under oxygen-free conditions. The obtained materials, after being cooled, were labeled as A&C300, A&C500 and A&C700 based on the temperature applied, respectively.

### 2.2. Characterization of Absorbent

The details of the methods and instruments applied in this study for adsorbent characterization are described in Text S1 of the Supplementary Materials.

### 2.3. Adsorption Experiments

The TC and Cu(II) stock solutions with a concentration of 1000 mg/L were obtained by dissolving 1 g of TC (C_22_H_24_N_2_O_8_, with purity above 98%) and 3.802 g of Cu(NO_3_)_2_·3H_2_O into 1 L of ultra-pure water.

For the adsorption performance test, 10 mg of adsorbents (A&C300, A&C500 and A&C700) were added into a 20 mL solution containing TC (10, 20, 50, 80, 100 and 120 mg/L) and Cu(II) (10, 20, 30, 40, 50 and 60 mg/L), respectively, followed by shaking under a constant temperature in the oscillation incubator at 25℃ with 180 rpm. The whole adsorption process underwent shading conditions and the initial pH of solutions was set at 6 to avoid the interference of H^+^ and OH^−^.

After shaking, samples were filtered by a 0.45 μm microporous membrane supplemented with a pump, and the residual concentration of TC and Cu(II) was determined by UV spectrophotometer (UV-1801, China) and ICP-OES (PerkinElmer Optima 2100DV, US); the standard curves for Cu(II) and TC are shown in Figure S1.

The removal rate and adsorption capacity were calculated by applying Equation (S1) and Equation (S2), respectively.

### 2.4. Adsorption Kinetics and Isotherms

The volume of reacted solution in the kinetics and isotherms experiments under single and binary combined pollution systems was set as 100 mL with un-adjusted pH, retaining the dosage of A&Cs at 100 mg.

For the kinetics, a fixed concentration of TC was set as 100 mg/L and the initial concentration of Cu(II) was set as 0, 10, 20, and 50 mg/L, respectively, thus the ratio of TC to Cu(II) in the binary system (BS) was initially kept as 10:1, 5:1, and 2:1 (Coded as BS-10, BS-5 and BS-2). Samples were obtained at 0.25, 0.5, 1, 2, 4, 6, 8, 12, 24 and 48 h as reserve. For the kinetics of Cu(II), the initial concentration was fixed as 50 mg/L, and the initial concentration of coexisting TC was set as 0, 5, 10 and 25 mg/L for the ratio of Cu: TC as 10:1, 5:1 and 2:1 in BS (Coded as BS-10, BS-5 and BS-2). The sampling time was consistent with that of the above step.

For the adsorption isotherms, the original concentrations of TC and Cu(II) were set as 10, 20, 50, 80, 100, 120 mg/L and 10, 20, 30, 40 and 60 mg/L, respectively. The concentration of coexisting Cu(II) (0, 10, 30 and 50 mg/L, coded as TC, TC+10Cu, TC+30Cu and TC+50Cu, respectively) on the TC adsorption isotherm and of coexisting TC (0, 5, 25, 50 mg/L, coded as Cu, Cu+5TC, Cu+25TC and Cu+50TC) on Cu adsorption isotherms were studied.

## 3. Results

### 3.1. Characterization of A&Cs

The basic physicochemical characteristics of A&C300, A&C500 and A&C700 were obtained and listed in Table S1. As seen, the yield of A&Cs dramatically declined from 84.34% to 70.3% when the applied pyrolysis temperature increased from 300℃ to 700℃. The pH value of A&Cs showed a reverse trend since the alkalinity from A&C300 to A&C700 gradually increased from 6.64 to 9.71. In addition, the ash contents of A&C300, A&C500 and A&C700 were 76.82, 84.41 and 87.15%, which might be attributed to the combustion of organic matters (oil residues) and the enrichment of mineral salts during the pyrolysis of SBE [23]. A positive effect of temperature for pH_pzc_ could be observed, as the pH_pzc_ values of A&C300, A&C500 and A&C700 were obtained as 6.20, 8.36 and 10.43, respectively (Figure S2). During the adsorption process, the protonation reaction caused positive charges covering the surface of A&Cs so as to immobilize anions when the solution pH is lower than the pH_pzc_ value. On the contrary, the deprotonation reaction would surround the A&C surface with negative charges and lead to adsorption for the cations when pH > pH_pzc_ [24].

**Figure 1:**
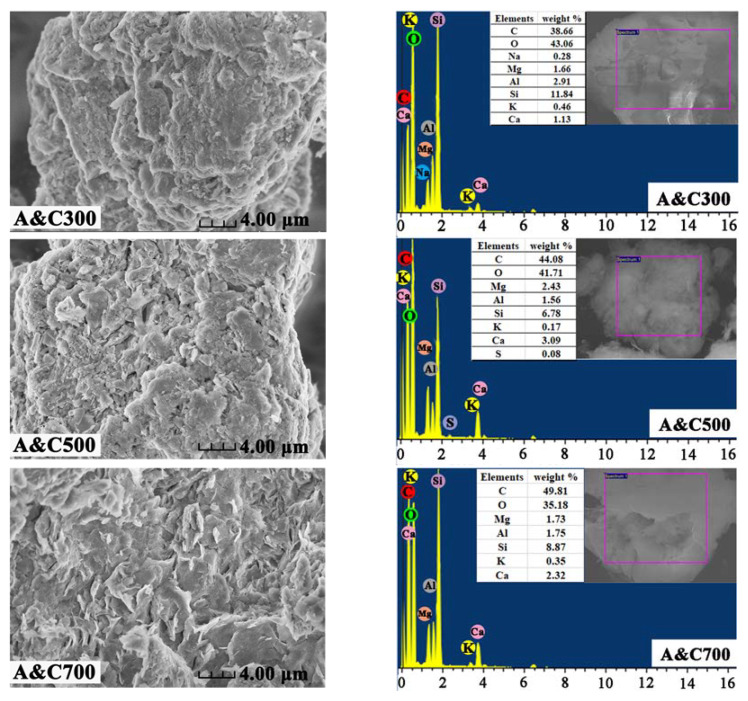
SEM-EDS images for A&C300, A&C500 and A&C700.

After pyrolysis, the porous structure can be re-observed from the morphology of A&Cs in SEM images. In Figure 1, the A&Cs contain a rough and uneven layered structure with irregular blocks and folds accumulated on the surface, which promoted the surface area to enhance the adsorption performance of A&Cs for pollutants. Notably, the lower pyrolysis temperature might play an appreciable role in removing the oil residues in A&Cs, resulting in the incomplete exposure of the porous structure for A&C300.

The major elements that existed on the surface of ACs were qualitatively analyzed and shown in EDS images (Figure 1). The presence of C mainly comes from the decomposition of oil residues and retained organic matter in spent bleaching earth during the pyrolysis process, and whose content exhibited an increasing trend under the higher pyrolysis temperature due to the enrichment effect. In addition, the detection of Al, Mg, O, Si and Ca in composites indicated the presence of attapulgite, since the attapulgite is mainly composed of clay mineral of magnesium aluminum silicate [25]. The above information offered evidence that the ACs successfully fabricated calcination, which aroused expectations for excellent performance in pollutant adsorption.

Generally, a developed porous structure played an important role in determining the adsorption performance of sorbent, as a larger specific surface area usually provides more active attachment sites for pollutants immobilization [26]. As can be seen from Table S1, the higher pyrolysis temperature facilitated the increase of specific surface area for A&Cs since the obtained specific surface areas of A&C300, A&C500 and A&C700 were 29.71, 77.73 and 79.24 m^2^/g, respectively. Furthermore, the higher pyrolysis temperature is also conducive to the formation of pores in A&Cs as the porous volume of A&C700 increased by nearly three times compared with A&C300 (from 0.063 cm^3^/g to 0.1741 cm^3^/g). However, the pore size of A&Cs only fluctuated in a certain limited range (from 43 to 44 nm), indicating that increased temperatures mainly promoted the formation of micropores and mesopores [27].

The crystal structure and composition of A&Cs were obtained from XRD diffraction plots (Figure S3). According to the diffraction patterns, the peaks in XRD can be classified into dispersion types due to the co-existence of weak peaks for amorphous diffraction and sharp peaks for crystal diffraction. The strong diffraction peaks in A&Cs at 2θ = 20.8°, 26.6°, 39.5°, 50.6°, 59.9° and 68.3° can be ascribed to the 100, 011, 102, 003, 121 and 031 crystal planes of SiO_2_ crystal, respectively, which means that all A&C300, A&C500 and A&C700 contained abundant silicon dioxide crystals. The characteristic peaks of attapulgite were observed at 2θ = 13.8°, 19.8°, 21.5°, 23.1°, 27.6°, 34.7° and 42.5° further proved the synthesis of A&C from spent bleaching earth [28,29,30,31]. In addition, it can be seen that the intensity of diffraction peaks tended to be stronger with the increase in applied pyrolysis temperature, which could be mainly attributed to the fact that the residues of oil and pigment attached to the SBE volatilized during the pyrolysis process so as to bear the crystals in SBE again (Figure S4). Therefore, the chaos degree of material becomes stronger and the structure tends to be more complete, which is conducive to the surface diffusion of pollutants due to the enhanced surface atomic activity [32]. Additionally, the XRD pattern also revealed that there might be more crystalline mineral components (e.g., CaCO_3_) and ash content existing on the surface of A&Cs, as quantities of small diffraction peaks were also detected [33].

Figure S5 exhibits the FTIR spectra of A&C300, A&C500 and A&C700, and the detailed functional groups corresponding to the peaks are listed in Table S2. The hydroxyl (-OH), alkenes (C=C) and carboxylic acid (C=O) were observed at 3435, 1634 and 1620 cm^−1^, respectively [34]. The aliphatic (C-H) poly-condensed from oils was detected at 1435 cm^−1^ in A&C300 and A&C500, but disappeared in A&C700, revealing the combustion of organic matters at excessive temperature. In addition, the FTIR results also displayed the dual characteristics of silicon- and carbon-based functional groups since A&Cs combined the attapulgite and functional groups induced by oil residue pyrolysis. The bands at 1039, 794 and 462 cm^−1^ could be attributed to the stretch of Si-O-Si, C-O-C, Si-O, C-O and Si-O-Al groups [35,36,37]. As the pyrolysis temperature grew, the volatilization of impurities and residues attached on SBE resulted in the polymerization of the carbon skeleton, which favored the formation of functional groups such as C=C, C=O, C-O-C; this result was also found in Zhang et al. (2022)—that higher temperature boosted the conversion of aliphatic into aromatic esters [38]. Nevertheless, the function groups formed would also tend to be destroyed under the exorbitant temperature [21]. In other words, a moderate temperature is more appropriate for A&Cs preparation when considering the comprehensive effects of pore dredging and functional group introduction.

### 3.2. Effect of Initial Concentration

**Figure 2:**
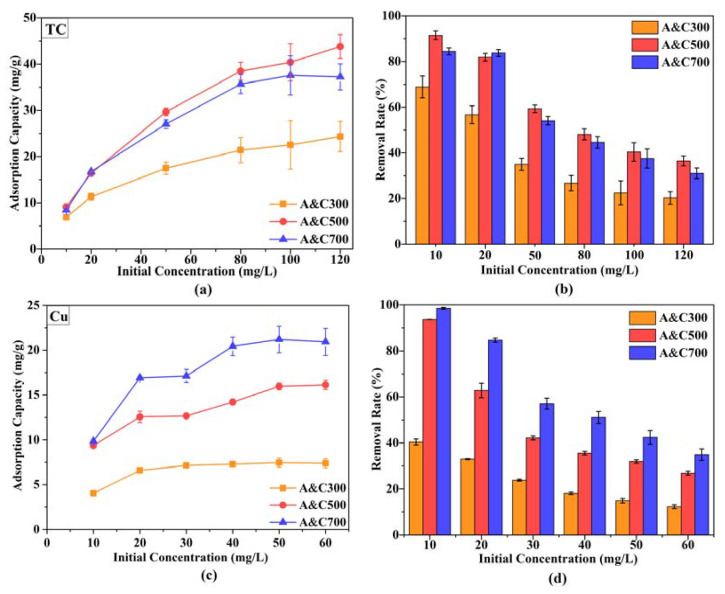
The adsorption performance and removal efficiency of A&Cs for (**a**,**b**) TC, and (**c**,**d**) Cu(II).

In the single system, the adsorption performance and removal efficiency of A&Cs for TC and Cu(II) under different initial concentrations are shown in Figure 2. As seen, the adsorption capacity of A&Cs for TC or Cu(II) significantly increased with the increase of Cu(II) or TC initial concentration, which might be due to the higher Cu(II) or TC concentration gradient between the liquid phase and adsorbent surface inhibiting the mass transfer resistance, thus promoting the adsorption reaction [39]. However, it cannot be ignored that the removal rate of Cu(II) or TC invariably displayed a negative correlation to initial concentration in Figure 2b,d, since the limited adsorption sites hardly accommodate excessive pollutants.

Furthermore, the pyrolysis temperature of A&Cs was recognized as a vital variable greatly influencing adsorption performance for TC and Cu(II). For TC, the adsorption amount under 120 mg/L of initial concentration was increased from 24.3 mg/g of A&C300 to 43.8 mg/g of A&C500 and then decreased to 37.2 mg/g of A&C700, suggesting that the pyrolysis temperature played an independent role in TC immobilization, and 500℃ was more appropriate for SBE preparation than 300℃ and 700℃ when applying A&Cs as the TC adsorbent. Because the trend of TC adsorption performance in A&C300, A&C500 and A&C700 showed a similar fluctuation with the intensity of surface functional groups in Figure S5, the complexation and hydrogen bond reaction may play a significant role in TC immobilization [40]. On the contrary, the Cu(II) adsorption performance of ACs followed an order of A&C700 > A&C500 > A&C300 as the adsorption capacity of A&C300, A&C500 and A&C700 at the 60 mg/L of initial Cu(II) concentration was 7.4, 16.1 and 20.9 mg/g, respectively, suggesting that the higher pyrolysis temperature was conducive to the adsorption sites formation for Cu(II) in A&Cs. In addition, it also evidenced by the alteration of the color intensity for Cu(II) solution from dark blue to light that the Cu(II) was successfully immobilized on A&Cs.

Overall, the A&C500 contained a relatively excellent adsorption performance for TC and Cu(II) compared with A&C700 and, taking the economic viability for practical application into consideration, the A&C500 (with lower energy consumption for preparation) was selected for further analysis.

### 3.3. Effect of Contact Time

**Figure 3:**
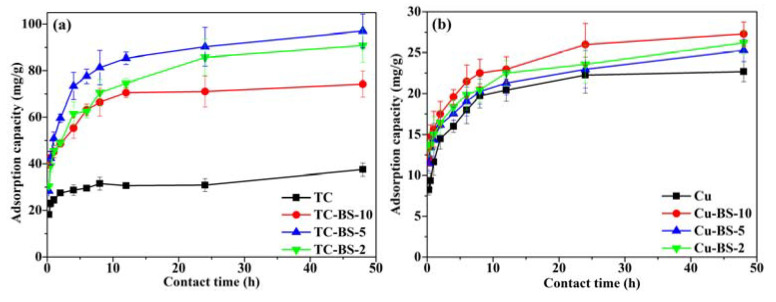
The adsorption performance of A&C500 for (**a**) TC and (**b**) Cu (II) under the single and binary systems as the function of contact time. (Note: the number in BS-10, BS-5 and BS-2 refers to the ratio between target adsorbate and competitive substance in a binary system (i.e., 10:1, 5:1 and 2:1)).

Only limited information about the adsorption potential of A&Cs for TC and Cu(II) has been provided from Section 3.2, and whether the product of re-generated SBE contains a value for practical application is also determined by its adsorption rate for TC and Cu(II), as the demanded time for saturating the adsorption sites in water treatment is an important parameter of an adsorbent [41]. Therefore, Figure 3 depicted the TC or Cu(II) adsorption capacity of A&C500 under single and binary systems (BS) at different contact times, in which the number in BS-10, BS-5 and BS-2 represents the ratio of target adsorbate and competitive substance in the binary system. It can be seen that the A&C500 breaks into the equilibrium stage for TC adsorption at approximately 8 h, while the required time under the competitive adsorption condition has been significantly prolonged. In addition, the adsorption capacity of A&C500 for TC at 48 h increased from 37.5 mg/g in the single system to 74.2 mg/g in TC-BS-10 and 97.0 mg/g in TC-Cu-5, suggesting that the presence of Cu(II) promoted the adsorption performance of A&C500 for TC. It should also be noticed, however, that the promoting effect of Cu(II) for TC immobilization does not offer a linear upward increase with Cu(II), as the TC adsorption capacity of A&C500 in TC-BS-2 declined 6.4% more than that in TC-BS-5, which might be ascribed to the competition effect for adsorption sites in A&C500 between redundant Cu(II) and TC under a binary system.

In contrast, the effect of TC in the binary system on Cu(II) is not so dramatic in Figure 3b since the Cu(II) adsorption capacity of A&C500 merely increased by 20.3% (Cu-BS-10), 11.5% (Cu-BS-5) and 15.4% (Cu-BS-2) than that in the single system, respectively. Meanwhile, the unchanged contact time for reaching the equilibrium stage under both single and binary systems strengthened the conclusion that TC posed limited effects on Cu(II) immobilization by A&C500. This phenomenon might result from the higher adsorption affinity of A&C500 for TC than Cu(II) under the competitive adsorption condition, which was in line with the results reported by Feng et al. (2022) [42].

### 3.4. Adsorption Kinetics

**Figure 4:**
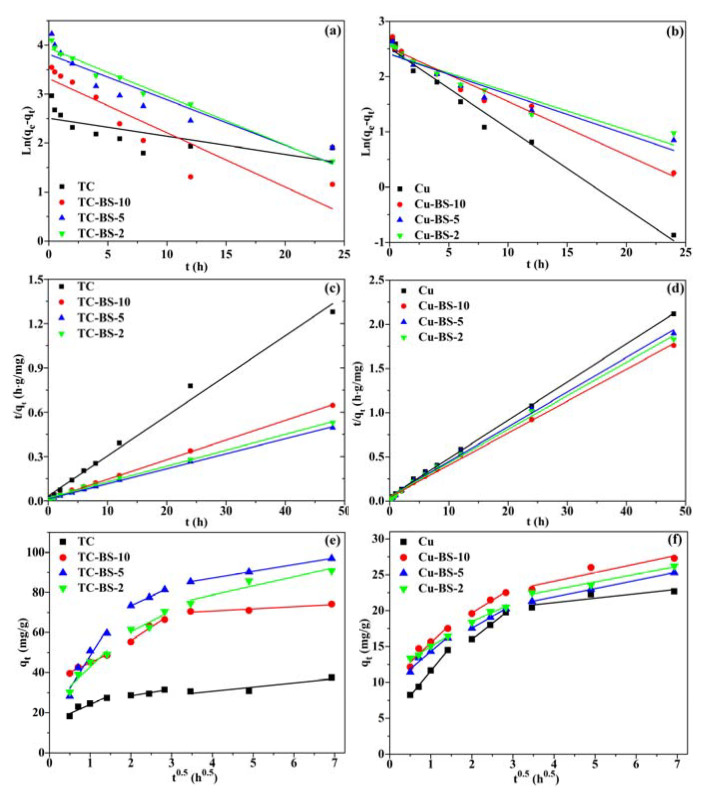
The fitted line of (**a**,**b**) pseudo-first-order kinetics model, (**c**,**d**) pseudo-second-order model; and (**e**,**f**) intra-particle diffusion model of A&C500 adsorbing of TC and Cu(II) under the single and binary systems.

Two commonly used kinetic models, pseudo-first (Equation (S3)) and pseudo-second-order models (Equation (S4)), were applied to offer some valuable insights related to the mechanisms of Cu(II) and TC adsorption by fitting the experimental data of A&C500 under the single and binary systems. The fitted line of the pseudo-first/pseudo-second-order kinetic models is exhibited in Figure 4a–d and the fitting parameters listed in Table S3, respectively. As seen, the *R*^2^ from pseudo-second-order kinetic model (0.989 in TC, 0.999 in TC-BS-10, 0.999 in TC-BS-5 and 0.996 in TC-BS-2; 0.999 in Cu, 0.998 in Cu-BS-10, 0.996 in Cu-BS-5, and 0.996 in Cu-BS-2) for TC and Cu(II) adsorption was higher than for pseudo-first-order model and the obtained fitting equilibrium adsorption capacity (*q*_e_) was closer to the actual adsorption capacity of A&C500 at 48 h (*q*_48h_), suggesting that the pseudo-second-order model was more appropriate in describing the whole adsorption process of A&C500 for TC or Cu(II) and chemical reaction might play a dominant role for TC and Cu(II) immobilization [43,44]. The constant of *K*_2_ from the pseudo-second-order kinetic model decreased from 0.023 of TC and 0.036 of Cu(II) in the single system to 0.006–0.016 (TC) and 0.027–0.029 (Cu-II) in the binary system, indicating that the dispersion of target pollutant on the surface of A&C500 has been repressed by the competitive disruptor, which follows the inferences from Figure 2 [45].

When the solid adsorbent falls into the reaction solutions, a liquid film that adheres to the surface of the adsorbent alters the mass transfer mode of TC and Cu(II) from the solution into A&Cs. Therefore, the liquid film diffusion controls the first step in the contact of pollutants and A&Cs, which depends on the mass transfer force inside and outside the liquid film rather than the adsorption performance of A&Cs. In addition, the pollutants inside the liquid film would then move into the adsorbent pores to be fixed by reacting with the active sites on A&Cs; this step is mainly driven by the disordered molecular thermal motion and has therefore been defined as intro-particle diffusion [27,46]. In order to identify the controlling step of TC and Cu(II) when transferring into A&C500 under the single and binary systems, the intra-particle diffusion model was adopted to simulate the adsorption data and the fitted lines are shown in Figure 4e,f and listed in Table S4. The fitted line of the whole adsorption process can be classified into three linear stages and each intercept of fitted line surpasses zero, suggesting that both liquid-film and intra-particle-diffusion processes limited the adsorption rate [47]. Moreover, the presence of Cu(II) promotes the mass transfer process of TC in the first step, since the *K*_i1_ value fitted in the TC-BS system was greater than that in the TC system.

### 3.5. Adsorption Isotherms

**Figure 5:**
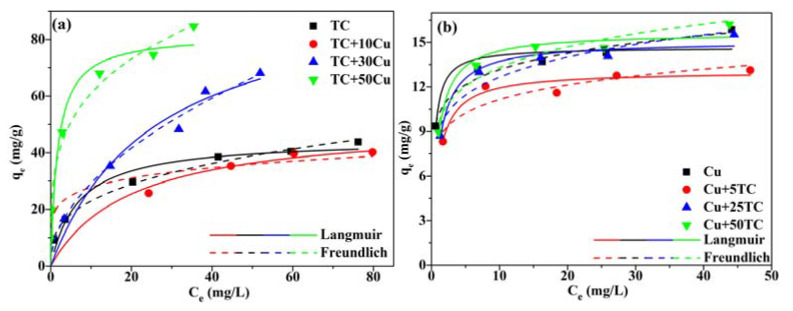
The isotherms of (**a**) TC and (**b**) Cu(II) fitted with Langmuir and Freundlich models under the single and binary system.

The behavior of A&C500 for TC and Cu(II) adsorption under the single and binary combined system at different initial concentrations has been investigated by fitting experimental data into Langmuir (Equation (S6)) and Freundlich (Equation (S7)) isotherm models and the corresponding fitted curves are described in Figure 5a,b. As seen from the parameters listed in Table S5, the higher coefficient (*R*^2^) of TC-fitted Freundlich isotherm models suggests that the Freundlich model was more appropriate in describing the TC adsorption isotherm than Langmuir in both single and binary systems, since a closer value to 1 of the determination coefficients represents a better fitting [48]. It can be speculated from this result that the TC was multi-layer adsorbed on the surface of A&C500, as the empirical equation of the Freundlich model established by assuming that the surface of adsorbate in the heterogeneous surface of adsorbent increased with the increase in pollutant concentration [49]. For Cu(II) adsorption isotherms, a different fitting result has been presented between single and binary systems. Under the single system, multi-layered adsorption occurred for Cu(II) adsorption as the Freundlich model obtained the higher *R*^2^ value, while for the binary system the Langmuir obtained a better description for the experimental data than the Freundlich model. The results indicate that Cu(II) was mainly fixed on A&C500 with mono-layered adsorption in the binary system, as the Langmuir model hypothesized that monolayered adsorption occurs at a specific homogeneous location in the adsorbent without interaction between adsorbate molecules [50]. Additionally, instead of competitive adsorption, the difference in adsorption capacity of Cu(II) and TC by A&C500 under the single and binary systems presents a synergistic effect between TC and Cu(II), in which TC is more sensitive to the co-existence of Cu(II) due to the higher adsorption capacity of TC in the binary system.

Table 1 lists the comparison of the theoretical maximum adsorption capacity for Cu(II) and TC between A&C500 obtained in this study and other adsorbents. Such adsorbents as biochar, cotton fiber, blast furnace slag, chitosan, et al., all failed in displaying higher adsorption potential for Cu(II) and TC compared to A&C500. However, it should also be noticed that there is still great room for improvement. Considering the low cost of A&C500 in preparation and its excellent performance in simultaneously removing organic and inorganic pollutants, A&C500 could act as a good precursor for further modification in order to obtain greater material for TC and Cu(II) remediation in subsequent research.

### 3.6. Competitive Adsorption Analysis

The distribution coefficient (*K*_d_) (Equation (1)) and selectivity coefficient (α) (Equation (2)) of A&C500 for TC and Cu(II) in the binary combined system were calculated to quantify the adsorption affinity of A&C500 for TC and Cu(II) in a mixed condition. *K*_d_ represents the proportion of target pollutants distributed in the solid and liquid phases at the equilibrium state and a higher value of *K*_d_ refers to a stronger adsorption affinity [59]. α indicates which adsorbate A&C500 tends to adsorb preferentially at the condition of co-existence of the interfering substance [60].

(1) $$K_{d}=\frac{V\cdot (C_{0}-C_{e})}{m\cdot C_{e}}$$

(2) $$\alpha =\frac{K_{d}(T)}{K_{d}(I)}$$

where *C*_0_ and *C*e (mg/L) are the concentrations of target pollutants at the initial and equilibrium state under the binary system; *m* (mg) refers to the mass of adsorbent; *V* (L) is the volume of solution, *K*_d_(*T*) (L/mg) is the *K*_d_ value of target pollutant, while *K*_d_(*I*) (L/mg) is the *K*_d_ value of the interfering substance.

The calculated *K*_d_ and α results of A&C500 when adsorbing Cu(II) and TC under initial concentrations of 10 and 50 mg/L are listed in Table 2. As seen, A&C500 contains a stronger adsorption affinity for TC than Cu(II). However, the *K*_d_(TC) and $\alpha_{\mathrm{Cu}}^{TC}$ values decreased significantly under the initial concentration at 50 mg/L, which means that the affinity and selectivity of A&C500 for TC has been inhibited by the higher concentration of the interfering substance. This result might be ascribed to the lower hydration radius of Cu(II), facilitating the diffusion process in contact with adsorption sites, so favoring its disadvantage in adsorption affinity.

### 3.7. Adsorption Mechanisms

The surface functional groups of A&C500 before and after adsorbing TC or Cu(II) under the single and binary systems were obtained and illustrated in Figure S6. It can be seen that the intensity of -OH at 3435 cm^−1^ decreased significantly after reacting with TC or Cu(II), which might be attributed to the formation of hydrogen bonding between the hydrogen bond donor of hydroxyl in TC and the protonated functional groups in TC, or the complexation reaction between Cu(II) and -OH [61,62,63,64]. Under the TC system (both single and binary conditions), the peaks of A&C500 at 1634 cm^−1^ shifted to 1621 cm^−1^ and 1624 cm^−1^, indicating that TC was immobilized on the surface of A&C500, so carbonyl (C=O) might act as a hydrogen bond acceptor to form hydrogen bonding with TC, since a tetracycline molecular contains 6 hydrogen bond donors and 10 hydrogen bond acceptors [65]. In contrast, the decreased intensity of C=O and C=C in A&C500 after adsorbing of Cu(II) might result from the π-π electron donor–accepter (EDA) interaction between C=C and metal cations or complexation of carboxyl with Cu(II) [66], while the changes in peak intensity at 1039 cm^−1^ and 794 cm^−1^ of A&C500 after reacting with TC and Cu(II) proved that C-O is involved in the adsorption process under single and binary systems. Additionally, the synergistic effect between TC and Cu(II) could be blamed on the π-π EDA interaction between Cu(II) and C=C in A&C500 that was introduced by immobilized TC [67,68].

As an adsorbent, A&C500 contains a relatively high specific surface area (77.729 m^2^/g) with abundant porous structure (0.1024 cm^3^/g) (Table S1), which provides sufficient space for the diffusion of Cu(II) and TC inside the internal surface of the adsorbent, realizing the capture of pollutants into the A&C500 matrix [68]. Meanwhile, it is well known that TC as an amphoteric substance contains three dissociation constants, which means that the electronic interaction might influence the A&C500 adsorption process for TC as the TC mainly acted as cations (H_4_TC^+^) when pH < 3.4 or existed as anions (H_2_TC^−^ or HTC^2−^) when pH > 7.6 [46,69]. However, the pH_pzc_ for A&C500 is 8.36 (Table S1), which was lower than the solution pH value at the equilibrium stage, indicates that the surface of A&C500 was mainly electroneutral or electronegative under the single and binary system. The electrostatic attraction did not participate in the adsorption for TC but contributed to the immobilization of Cu(II).

**Figure 6:**
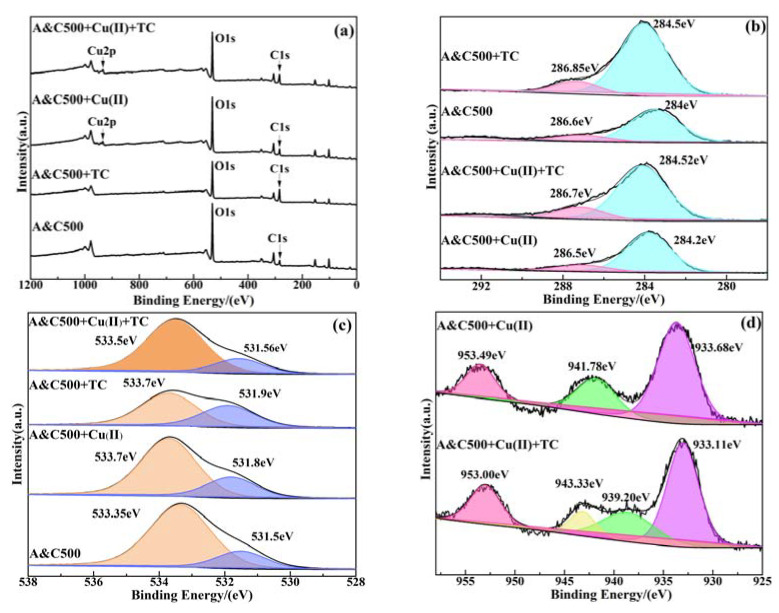
The XPS spectrum of A&C500 before and after adsorbing of TC and Cu(II), (**a**) full-spectrum; (**b**) C1s; (**c**) O1s; (**d**) Cu2p.

The tested XPS spectrum of A&C500 before and after adsorbing of TC and Cu(II) is illustrated in Figure 6, and the corresponding elements’ chemical properties are listed in Table S6. From the full-spectrum (Figure 6a), it is clear that the peaks of Cu2p appeared on the A&C500 after reacting with Cu(II), while the intensity of C1’s peak is significantly enhanced after reacting with TC, indicating that TC and Cu(II) were adsorbed on the surface of A&C500 successfully. However, the intensity of O1s detected in A&C500 decreased significantly under the single and binary systems, suggesting that oxygen-containing functional groups were completely engaged in the adsorption process. Meanwhile, the peaks at 284 eV and 286.6 eV corresponding to C-H/C=C and C-O in the C1s spectrum (Figure 6b) as well as the peaks at 533.35 eV of O-H/C-O-C and 531.5 eV of C=O in O1s under the single system all shifted from the original position, which represents an affinity between TC/Cu(II) and functional groups. This result was also consistent with the changes in the spectrums of FTIR. In addition, the Cu(II) could act as a bridge to bind with C=O and -OH in TC to form a TC-Cu(II) complex compound under the binary system after reacting with the adsorbent, resulting in a greater shift for peak positions of O-H/C-O-C and C=O in A&C500 [70]. This explained why A&C500 obtained higher TC adsorption performance in the binary system and the transformation of Cu(II) adsorption from multi-layered in the single system into mono-layered adsorption in the binary system (Table S5). The bridging effect of Cu(II) and TC were also exhibited in the position variation of Cu2p peaks from 933.68, 941.78, and 953.49 eV in A&C500+Cu(II) system to 933.11, 939.20, and 953.00 eV in A&C500 under A&C500+TC+Cu(II) system (Figure 6d). Additionally, the new peak of Cu2p at 943.33 eV under the binary system can be mainly attributed to the complex compound of Cu(II)-TC [70,71].

To conclude, the mechanisms of A&C500 for TC adsorption mainly include pore-filling effect, hydrogen bonding and-π EDA interaction, while the adsorption for Cu(II) could be mainly attributed to the pore-filling effect, electrostatic attraction, π-π EDA interaction and complexation, respectively. Figure S7 exhibits the adsorption process between A&C500 and TC/Cu(II) under single/binary systems.

## 4. Conclusions

A novel adsorbent (A&C) was prepared by pyrolyzing spent bleaching earth (SBE) for the synergetic removal of TC and Cu(II) in aquaculture wastewater. Under three different pyrolysis temperatures (300, 500 and 700 °C), A&C500 achieved the highest potential for practical application due to its excellent adsorption performance with lower energy consumption. Compared with Cu(II), A&C500 exhibited higher adsorption affinity for TC and the TC adsorption performance has been further promoted by Cu(II) under the binary system, showing a synergistic effect between TC and Cu(II). In addition, the kinetic analysis suggested that chemical adsorption played the dominant role in the removal of TC and Cu(II), in which the diffusion (both liquid-film diffusion and intra-particle diffusion) process controlled the whole adsorption rate. The characterization of A&C500 before and after adsorption revealed that bridging effects between TC and Cu(II) strengthened the adsorption performance of A&C500 for TC under binary system, which was conducive to the synergistic removal of TC and Cu(II) in wastewater. In addition, pore-filling, hydrogen bond, π-π EDA, electrostatic attraction and complexation are also involved in the removal of TC and Cu(II). This study provides new insights into the utilization of transformed SBE as an adsorbent with synergistic removal of TC and Cu(II) from aquaculture wastewater.

## Figures and Tables

**Table 1:** Comparison of adsorption capacity for Cu(II) and TC by different adsorbents.

Adsorbent	Cu(II) (mg/g)	TC (mg/g)	References
A&C500	14.70	44.90	This study
Swine manure biochar	8.98		[51]
Cotton fiber	6.12		[52]
Blast furnace slag	8.49		[53]
Residue & soil adsorption composite	2.63		[54]
Sludge-derived adsorbent		30.00	[55]
Graphene oxide functionalized magnetic particles		39.10	[56]
Oak biochar		11.85	[57]
Chitosan		31.00	[58]

**Table 2:** The parameters of distribution and selectivity of A&C500 for Cu and TC under the binary system.

	*C*_0_	*K*_d_ (TC)	*K*_d_ (Cu)	$\alpha_{\mathrm{Cu}}^{TC}$	$\alpha_{\mathrm{TC}}^{Cu}$
A&C500	10	249.376	7.418	33.620	0.030
	50	16.585	0.929	17.845	0.056

## Data Availability

The data presented in this study are available upon request from the corresponding author.

## Supplementary Materials

The following supporting information can be downloaded at: https://www.mdpi.com/article/10.3390/ijerph20021573/s1, Figure S1: Standard curves for Cu(II) and TC; Figure S2: The pH at point of zero charges (pHpzc) for A&C300, A&C500 and A&C700; Figure S3: X-ray diffraction plots of A&C300, A&C500 and A&C700; Figure S4: The pyrolysis process of SBE; Figure S5: The FTIR spectrums of A&C300, A&C500 and A&C700; Figure S6: The FTIR spectroscopy of A&C500; Figure S7: Adsorption mechanisms of Cu(II) and TC on A&C500; Table S1: Physicochemical properties of A&Cs; Table S2: Main functional groups observed for A&Cs; Table S3: The fitted parameters of pseudo-first-order and pseudo-second-order kinetic model; Table S4: The fitted parameters of intra-particle diffusion model; Table S5: The fitted parameters of Langmuir and Freundlich isotherm model; Table S6: Binding energy lookup table for signals from C1s, O1s and Cu2p of A&C500.
