# Supplementary material for: A Novel Adsorbent of Attapulgite & Carbon Composites Derived from Spent Bleaching Earth for Synergistic Removal of Copper and Tetracycline in Water
Source: Int J Environ Res Public Health. 2023 Jan 15;20(2):1573. doi: 10.3390/ijerph20021573 (PMC9865348; doi:10.3390/ijerph20021573)
Supplement: Supplementary file 1 [file ijerph-20-01573-s001.zip › ijerph-2105638-supplementary.pdf]

*Supplementary Material*

# A Novel Adsorbent of Attapulgite & Carbon Composites Derived from Spent Bleaching Earth for Synergistic Removal of TC and Cu(II) in Water

**Yuxin Ke [1, 2], Xiaoli Zhu [1, 2, *], Shaocheng Si [1, 2], Ting Zhang [1, 2], Junqiang Wang [1, 3] and Ziye Zhang [3]**

1 College of Urban and Environmental Science, Northwest University, Xi'an, 710127, China

2 Shaanxi Key Laboratory of Earth Surface System and Environment Carrying Capacity, Xi'an, 710127, China;

3 Xi'an Jinborui Ecological Tech. Co., Ltd. Xi'an, 710065, China

* Correspondence: xiaolizhu@nwu.edu.cn

**Text S1**

The characterization corresponding to mechanism analysis of A&Cs was conducted as follows. The micro-morphology, structure and surface elemental composition of A&Cs were analyzed by SEM-EDS (HITACHI SU8010, Japan). The specific surface area was calculated through the BET method, while FTIR (BRUKER Invenio, Germany) and XPS (ULVAC -PHI - 5000VPIII, Japan) were applied to investigate the chemical states of surface elements and the functional groups of A&Cs. The XRD (Bruker D8, Germany) was used to reflect the types and structure of crystals in A&Cs. The determination of pHpzc (the pH value for sorbent at the point of zero charges) for A&Cs were conducted through the pH drift method, in which the pH values were measured by the pH meter (Mettler Toledo FE28, Switzerland).

**Eq. S1 Removal efficiency:**

$$\eta = \frac{C_0 - C_e}{C_0} \times 100\% \tag{S1}$$

**Eq. S2 Adsorption capacity:**

$$Q_e = \frac{C_0 - C_e}{m} \times V \tag{S2}$$

Where $\eta$ (%) and $Q_e$ (mg/g) represent removal rate and the adsorption capacity at equilibrium state, respectively; $C_0$ and $C_e$ (mg/L) are the concentration before and after adsorption, respectively.

**Eq. S3 Pseudo-first-order (PFO) kinetic model:**

$$Ln(q_e - q_t) = Ln(q_e) - K_1 t \tag{S3}$$

**Eq. S4 Pseudo-second-order (PSO) kinetic model:**

$$\frac{t}{q_t} = \frac{1}{K_2 q_e^2} + \frac{t}{q_e} \tag{S4}$$

Where $q_t$ and $q_e$ (mg/g) are the adsorption capacity of A&C500 for TC or Cu(II) at the time $t$ (h) and equilibrium stage; $K_1$ ($min^{-1}$) and $K_2$ (g/(mg·min)) refer to the constant of pseudo-first-order and pseudo-second-order kinetic model, respectively.

**Eq. S5 Intra-particle diffusion (IPD) kinetic model:**

$$q_t = K_i \cdot t^{0.5} + C_i \tag{S5}$$

Where $K_i$ (mg/(g·h)) refers to the constant of intra-particle diffusion kinetic model; and $C_i$ is the constant corresponding to the thickness of the boundary layer.

**Eq. S6 Langmuir isotherm model:**

$$q_e = \frac{q_m K_L C_e}{1 + K_L C_e} \tag{S6}$$

**Eq. S7 Freundlich isotherm model:**

$$q_e = K_F C_e^{\frac{1}{n}} \tag{S7}$$

Where $q_e$ (mg/g) is the adsorption capacity of TC or Cu(II) at equilibrium state, $q_m$ (mg/g) is the maximum sorption capacity of A&C500 for TC or Cu(II); KL (L/mg) is the Langmuir constant that relates to the free energy of sorption; $K_F$ is the Freundlich constant related to sorption capacity; $n$ is an empirical parameter which varies with the degree of heterogeneity of adsorbing sites.

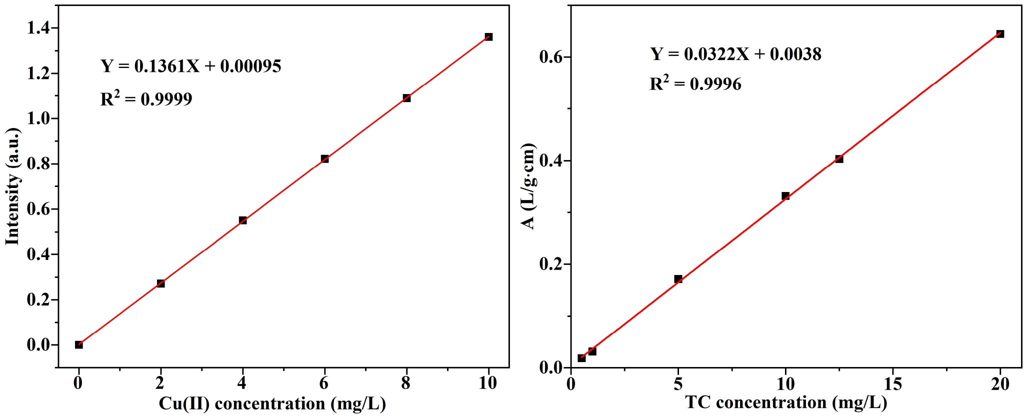


**Figure S1** Standard curves for Cu(II) and TC

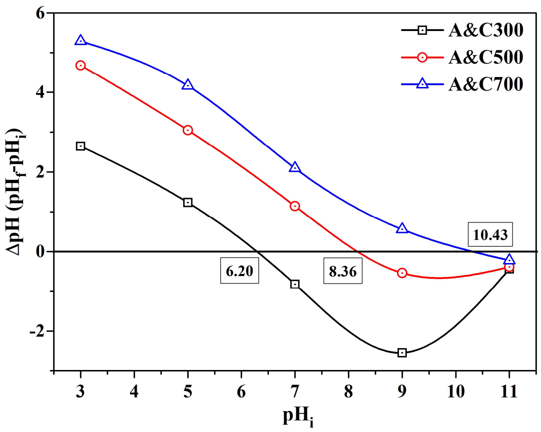


**Figure S2** The pH at point of zero charges ($pH_{pzc}$) for A&C300, A&C500 and A&C700.

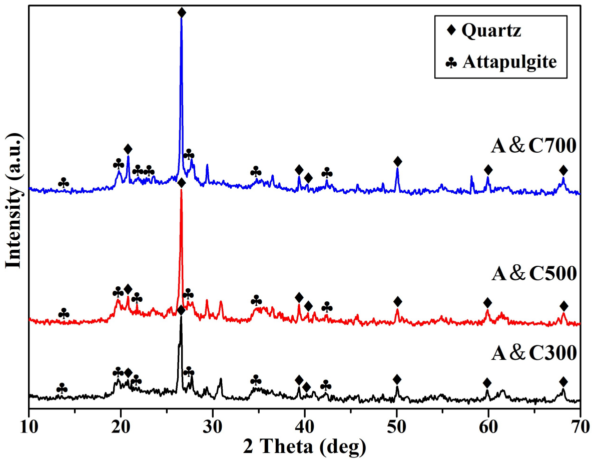


**Figure S3** X-ray diffraction plots of A&C300, A&C500 and A&C700.

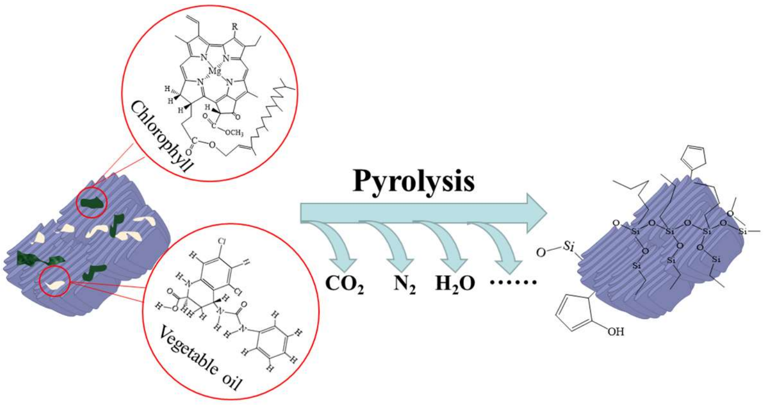


**Figure S4** The pyrolysis process of SBE.

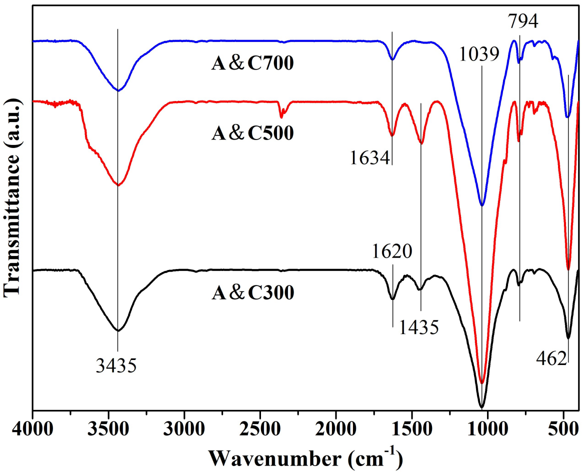


**Figure S5** The FTIR spectrums of A&C300, A&C500 and A&C700.

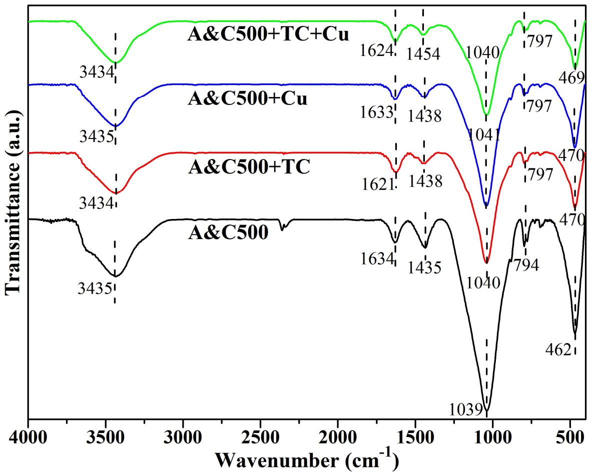


**Figure S6** The FTIR spectroscopy of A&C500.

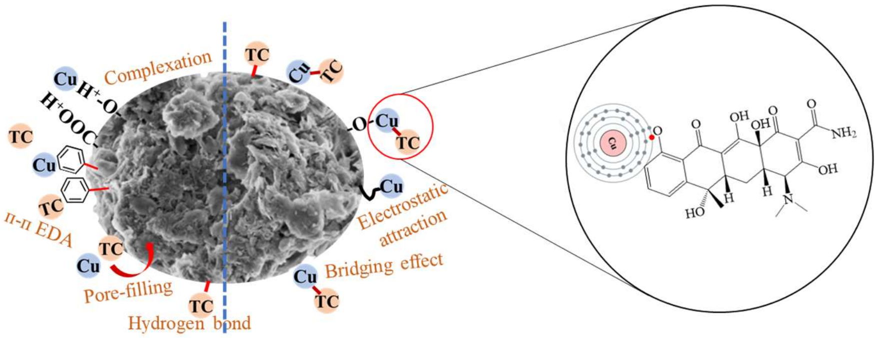


**Figure S7** Adsorption mechanisms of Cu(II) and TC on A&C500

**Table S1** Physicochemical properties of A&Cs

| Adsorbent | Specific surface area | Total volume | Average pore size | pH | Ash content | Yield | $pH_{pzc}$ |
|---|---|---|---|---|---|---|---|
| | $m^2/g$ | $cm^3/g$ | nm | | % | % | |
| A&C300 | 29.71 | 0.0630 | 43.660 | 6.64 | 76.82 | 84.34 | 6.20 |
| A&C500 | 77.729 | 0.1024 | 43.754 | 9.03 | 84.41 | 74.02 | 8.36 |
| A&C700 | 79.24 | 0.1741 | 43.944 | 9.71 | 87.15 | 70.30 | 10.43 |

**Table S2** Main functional groups observed for A&Cs

| | | | | | | | | |
|---|---|---|---|---|---|---|---|---|
| A&C500 | Wavenumber ($cm^{-1}$) | 3435 | 1634 | 1620 | 1435 | 1039 | 794 | 462 |
| | Functional groups | -OH | C=C | C=O | C-H | C-O-C<br>Si-O-Si | Si-O<br>C-O | Si-O-Al |

**Table S3** The fitted parameters of pseudo-first-order and pseudo-second-order kinetic model

| | Pseudo-first-order kinetic model | | | | Pseudo-second-order kinetic model | | | |
|---|---|---|---|---|---|---|---|---|
| | $q_e$ | $q_{48h}$ | $K_1$ | $R^2$ | $q_e$ | $q_{48h}$ | $K_2$ | $R^2$ |
| | mg/g | mg/g | $min^{-1}$ | | mg/g | mg/g | g/(mg·min) | |
| TC | 12.3 | 37.5 | 0.037 | 0.439 | 36.9 | 37.5 | 0.023 | 0.989 |
| TC-BS-10 | 27.5 | 74.2 | 0.111 | 0.821 | 75.0 | 74.2 | 0.016 | 0.999 |
| TC-BS-5 | 45.2 | 97.0 | 0.093 | 0.834 | 98.2 | 97.0 | 0.008 | 0.999 |
| TC-BS-2 | 51.3 | 90.8 | 0.099 | 0.978 | 92.9 | 90.8 | 0.006 | 0.996 |
| Cu | 12.3 | 22.7 | 0.145 | 0.981 | 23.2 | 22.7 | 0.036 | 0.999 |
| Cu-BS-10 | 12.4 | 27.3 | 0.097 | 0.964 | 27.7 | 27.3 | 0.029 | 0.998 |
| Cu-BS-5 | 11.1 | 25.3 | 0.072 | 0.916 | 25.5 | 25.3 | 0.028 | 0.996 |
| Cu-BS-2 | 11.1 | 26.2 | 0.069 | 0.901 | 26.4 | 26.2 | 0.027 | 0.996 |

Note: The qe refers to the modeled equilibrium adsorption capacity of A&C500 for TC or Cu(II) through pseudo-first-order kinetic model, while the $q_{48h}$ is the adsorption capacity of A&C500 at the contact time of 48h.

**Table S4** The fitted parameters of intra-particle diffusion model

| | $K_i1$ | C1 | $R^2$ | $K_i2$ | C2 | $R^2$ | $K_i3$ | C3 | $R^2$ |
|---|---|---|---|---|---|---|---|---|---|
| | mg/(g·h) | | | mg/(g·h) | | | mg/(g·h) | | |
| TC | 9.182 | 14.929 | 0.842 | 3.405 | 21.630 | 0.839 | 2.084 | 22.378 | 0.702 |
| TC-BS-10 | 9.608 | 35.305 | 0.971 | 13.481 | 29.001 | 0.927 | 1.102 | 66.308 | 0.835 |
| TC-BS-5 | 32.393 | 15.933 | 0.891 | 9.544 | 54.284 | 0.999 | 3.367 | 73.713 | 0.999 |
| TC-BS-2 | 19.507 | 23.307 | 0.839 | 10.594 | 39.167 | 0.594 | 4.545 | 60.538 | 0.811 |
| Cu | 6.954 | 4.637 | 0.996 | 4.493 | 7.002 | 0.999 | 0.621 | 18.617 | 0.628 |
| Cu-BS-10 | 5.422 | 10.089 | 0.888 | 3.558 | 12.558 | 0.971 | 1.209 | 19.253 | 0.792 |
| Cu-BS-5 | 4.857 | 9.433 | 0.933 | 3.261 | 11.035 | 0.999 | 1.160 | 17.262 | 0.999 |
| Cu-BS-2 | 3.479 | 11.514 | 0.987 | 2.559 | 13.358 | 0.930 | 1.089 | 18.544 | 0.960 |

**Table S5** The fitted parameters of Langmuir and Freundlich isotherm model

| | Langmuir isotherm model | | | Freundlich isotherm model | | |
|---|---|---|---|---|---|---|
| | $q_m$ | $K_L$ | $R^2$ | $n$ | $K_F$ | $R^2$ |
| TC | 44.9 | 0.148 | 0.949 | 3.058 | 10.8 | 0.991 |
| TC+10Cu | 50.9 | 0.050 | 0.213 | 6.289 | 19.2 | 0.886 |
| TC+30Cu | 99.0 | 0.039 | 0.921 | 1.965 | 9.0 | 0.948 |
| TC+50Cu | 59.2 | 0.575 | 0.936 | 3.814 | 33.3 | 0.986 |
| Cu | 14.7 | 2.629 | 0.847 | 8.621 | 10.0 | 0.961 |
| Cu+10TC | 13.1 | 1.051 | 0.914 | 8.264 | 8.4 | 0.802 |
| Cu+25TC | 15.1 | 1.006 | 0.954 | 6.849 | 9.0 | 0.906 |
| Cu+50TC | 15.6 | 1.182 | 0.946 | 6.993 | 9.5 | 0.910 |

**Table S6** Binding energy lookup table for signals from C1s, O1s and Cu2p of A&C500

| Elements | | A&C500 | A&C500+TC | A&C500+Cu(Ⅱ) | A&C500+TC+Cu(Ⅱ) |
|---|---|---|---|---|---|
| | | Binding Energy | | | |
| C1s | C-H/C=C | 284 | 284.5 | 284.2 | 284.52 |
| | C-O | 286.6 | 286.85 | 286.5 | 286.7 |
| O1s | O-H/C-O-C | 533.35 | 533.7 | 533.7 | 533.5 |
| | C=O | 531.5 | 531.9 | 531.8 | 531.56 |
| Cu2p | Cu2p3/2 | | | 933.68 | 933.11 |
| | | | | 941.78 | 939.20 |
| | | | | - | 943.33 |
| | $Cu2p_{1/2}$ | | | 953.49 | 953.00 |
